# Research on a Task Offloading Strategy for the Internet of Vehicles Based on Reinforcement Learning

**DOI:** 10.3390/s21186058

**Published:** 2021-09-09

**Authors:** Shuo Xiao, Shengzhi Wang, Jiayu Zhuang, Tianyu Wang, Jiajia Liu

**Affiliations:** 1 School of Computer Science and Technology, China University of Mining and Technology, Xuzhou 221000, China; sxiao@cumt.edu.cn (S.X.); ts20170045a31@cumt.edu.cn (S.W.); ts20170072p31@cumt.edu.cn (T.W.); 2 Agricultural Information Institute, Chinese Academy of Agricultural Sciences, Beijing 100080, China; liujiajia@caas.cn; 3 Key Laboratory of Agri-Information Service Technology, Ministry of Agriculture, Beijing 100080, China

**Keywords:** Internet of Vehicles, mobile edge computing, task offloading, Stackelberg game, reinforcement learning

## Abstract

Today, vehicles are increasingly being connected to the Internet of Things, which enables them to obtain high-quality services. However, the numerous vehicular applications and time-varying network status make it challenging for onboard terminals to achieve efficient computing. Therefore, based on a three-stage model of local-edge clouds and reinforcement learning, we propose a task offloading algorithm for the Internet of Vehicles (IoV). First, we establish communication methods between vehicles and their cost functions. In addition, according to the real-time state of vehicles, we analyze their computing requirements and the price function. Finally, we propose an experience-driven offloading strategy based on multi-agent reinforcement learning. The simulation results show that the algorithm increases the probability of success for the task and achieves a balance between the task vehicle delay, expenditure, task vehicle utility and service vehicle utility under various constraints.

## 1. Introduction

The past years have seen a growth in mobile communication, chip technology, sensor technology and artificial intelligence. Vehicles are becoming more intelligent by installing advanced sensors, such as cameras and LIDAR. These vehicles are no longer limited to human control, but gradually become an intelligent and connected computing system. Such vehicles are called intelligent and connected vehicles (ICV) [1]. ICV are equipped with high-precision sensors and other computing devices such as powerful CPU and GPU, and can realize the information exchange and resource sharing of vehicle-to-vehicle (V2V), vehicle-to-pedestrian (V2P) and vehicle-to-infrastructure (V2I). The ICV industry [2] is based on artificial intelligence, big data, cloud computing and 5G communication technology, and supported by electronic information industry, communication industry, integrated circuit industry, internet industry, transportation industry; it is applied to the Internet of Vehicles, autopilot and market service industry. Based on the automation degree of the autopilot system, the Society of Automotive Engineers (SAE) divides autopilot into six levels (L0–L5). From level 0 to level 5 is a process of human-driven to machine-driven. In level 5, all driving operations of a driver are performed by the autopilot.

There are two modes of ICV, one is autonomous vehicle, the other is connected vehicle [3]. As an autonomous vehicle is equipped with advanced sensors and computing devices, it has situational awareness. Autopilot of the vehicle can analyze the driving state through on-board computing unit. The connected vehicle uses the next generation of mobile communication technologies to establish V2V, V2I, and V2P connections. Autopilot of the connected vehicle obtains external environmental data to assist vehicle control [4]. European union countries focus on developing autonomous vehicles; they attempt to achieve high-level autonomous driving by enhancing the perception and computing power of a single vehicle. However, as it is difficult to balance the environmental sensor’s accuracy and price, and the lack of reliability and security of related algorithm, the development of this mode has entered a bottleneck period. To solve the problems of autonomous vehicles, it is more and more important to explore the cooperative vehicle infrastructure system by installing sensors, communication devices, and edge computing nodes on road sections to improve the accuracy and range of vehicle perception, enhance computing power, and improve the reliability of autopilot.

In recent years, with the rapid popularization of ICV, road traffic density has greatly increased [5,6]. Computing resources of ICV are limited and cannot meet the huge amount of data generated by autopilot; uploading data to a remote cloud server results in high time delays and additional communication costs. Therefore, it is a problem to make the intelligent connected vehicle meet the requirements of low time delay and provide users with a high quality experience. The existing solution is to offload the task to edge cloud servers [7]. In IoV, vehicles can offload tasks to edge servers or other vehicles to accelerate task processing. However, moving vehicles cause communication interruptions during task offloading, and unstable long-distance communication between vehicles and cloud servers greatly increases the time delay. This environment cannot meet the needs of time-sensitive tasks. In recent years, as an improved resource allocation architecture of cloud computing, mobile edge computing has been introduced into the IoV to improve vehicles’ computing power during task processing [8].

With the development of 5G technology, an increasing number of vehicles have been connected to the IoV, and computing resources have become increasingly scarce. Energy consumption should have a low priority of intelligent connected vehicles that have sufficient power [9]. In view of this, we give priority consideration to the utilization of computing resources and propose a task offloading strategy based on deep reinforcement learning. The novelty and major contributions of this paper include the following:

1. Based on the Stackelberg game, we propose a new task offloading algorithm with deep reinforcement learning. It is different from previous research on minimizing task delays and power consumption. The goal of optimization is to reach a balance between time delay and task cost.
2. We conduct experiments and solve the Nash equilibrium between service vehicles and task vehicles.
3. We compare the proposed method with other algorithms. Our proposed algorithm achieves high system utility and better performance.

The rest of the paper is organized as follows. Section 2 introduces the related work of our research. In Section 3, we introduce the system framework, communication and cost model of this paper. In Section 4, the calculation model and utility function of the service vehicle are presented. In Section 5 and Section 6, we describe the algorithm and analyze the simulation results, respectively. Finally, the conclusion is drawn in Section 7.

## 2. Related Work

At present, many researchers and manufacturing companies have carried out related research work in the field of IoV [10,11]. Raza et al. [12] proposed the framework of vehicle edge computing (VEC). Using artificial intelligence technology, the author of [13] proposed a method of V2V communication to maximize the vehicle traffic flow in the transport system. Si et al. [14] proposed a solution to utilize the potential resources of vehicles in the Internet to solve the congestion problem in other data networks. To solve the problem of limited computing resources of edge servers, Zhang et al. [15] introduced a backup server to expand computing capacity and proposed a vehicular offloading framework. To balance the average delay and the server load, a dynamic service allocation algorithm based on Pareto optimality was proposed by Hu et al. [16] that solved the problem of edge node assignment. To expand the available cloud services for vehicle mobile applications, Li et al. [17] proposed a framework that combines a long-distance cloud server, edge computing node and vehicular cloud service. Huang et al. [18] proposed a reputation management system based on a vehicular network in which the allocation of computing resources was determined by the reputation of users. Liu et al. [19] introduced game theory into vehicle edge calculation, modeled task offloading as a multi-user non-cooperative game, and proved the existence of a Nash equilibrium solution in the game. Because wireless channel resources are limited, compared with binary offloading, it is more reasonable for us to use partial offloading [20,21,22,23].

In addition, the Doppler effect caused by the high-speed movement of vehicles, the shielding of peripheral objects on the road, and the communication interruption caused by the movement of edge nodes all lead to frequent changes in the topology of IoVs. To improve service quality, Hou et al. [20] proposed a method that used vehicles with residual resources as communication nodes and computing nodes. In [24], factors such as resource limitations and delay tolerances of IoV were taken into account, and K. Zhang et al. proposed a computing resource allocation model based on contract theory to maximize the benefits of edge server providers. Ren et al. [25] proposed a new partial offloading model in which part of the data is processed locally and the other part is processed on the edge nodes. This model improved the resource utilization of local and edge nodes and reduced the computing delay. In [26], Luoto Pet al. proposed a task offloading model with a low signal-to-noise ratio for IoV connected to roadside units (RSU) by combining V2I and V2V communication. To realize the joint optimization of servers and vehicles, Zhang et al. [27] proposed an effective combination mode of delay prediction in which computing tasks were transmitted to edge nodes through V2I or V2V communication.

In summary, the results of recent research on task offloading architecture can be divided into three methods: vehicle-to-vehicle architecture, vehicle-to-edge server architecture, vehicle-to-edge server and cloud server architecture. The vehicle-to-vehicle architecture was proposed to solve the cooperation problem among vehicles. To fully utilize the computing power of moving vehicles, the vehicle-to-edge server architecture was designed. The vehicle-to-edge server and cloud server architecture was proposed to realize a balance among vehicles, edge servers and cloud servers. To optimize the task delay, task energy consumption, load balancing of edge nodes and task completion probability, an intelligent optimization algorithm, contract strategy, game strategy, reinforcement learning algorithm [28,29,30], etc., are used to solve the offloading problem of moving vehicles.

## 3. System Model

**Figure 1:**
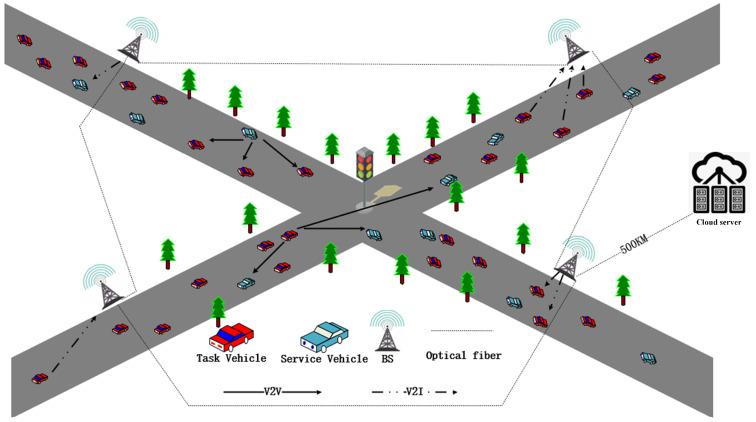
Task offloading system model based on Stackelberg–MADDPG algorithm.

Suppose that there are two roads, the lengths of which are defined as $L_{{road}_{-}h}$ and $L_{{road}_{-}v}$, respectively, and the widths as $L_{width}$. These two roads are perpendicular. Four RSUs are located in each road. These RSUs are connected to each other through optical fibres. To save on construction costs, only one RSU can connect to the cloud server [31]. Vehicles are randomly distributed at the crossroads. In each time slot, vehicles that are idle in the system have a probability of generating computing tasks. Vehicles with computing tasks are called task vehicles, while vehicles without computing tasks and with rich computing resources are called service vehicles. Vehicles at the crossroads can choose to go straight, wait, turn left or right. Vehicles in other positions continue to move in a straight line. If the distance between the task vehicle and the service vehicle is less than the V2V distance, then V2V communication is used. If the distance between the task vehicle and the service vehicle exceeds the V2V distance, V2I communication is used. The system model is shown in Figure 1.

**Figure 2:**
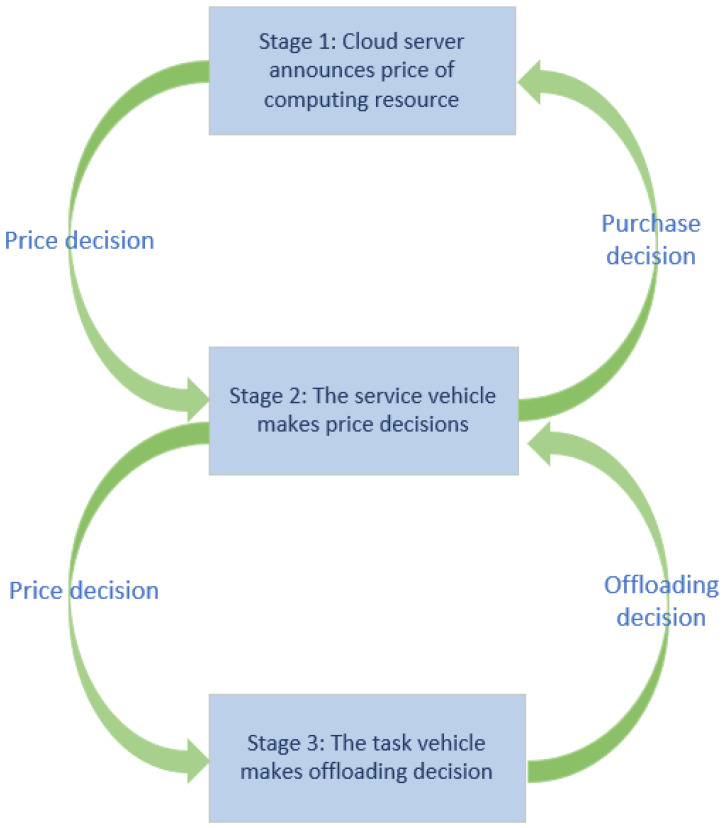
Three-stage Stackelberg game model.

As shown in Figure 2, this paper proposes a three-stage Stackelberg game model. In the first stage, the cloud server broadcasts the unit price of its computing power to the service vehicle according to its maximum computing power. In the second stage, the service vehicle determines the unit price of its computing power based on its current CPU utilization rate and the unit computing power price of the cloud server. Then, the service vehicle broadcasts its unit price of computing power to other task vehicles. In the third stage, the task vehicle chooses offloading strategies based on its price sensitive factor, delay sensitive factor, task priority, task success sensitivity factor and task information.

### 3.1. Communication Model

There are two communication modes in this system: V2I communication between vehicles and infrastructure and V2V communication between vehicles. V2V communication uses orthogonal frequency to enable vehicles to transmit without interference. This adopts the simple independent same-distributed channel. According to [26], the path loss PL of V2V communication at time *t* can be calculated as Equation (1):

(1) $$PL_{V2V}\left(t\right)=63.3+17.7\log_{2}d_{i,j}\left(t\right),$$

where $d_{i,j}\left(t\right)$ denotes the distance between vehicles $v_{i}$ and $v_{j}$ at time *t*. Assuming that the value of noise is 0, the communication bandwidth is denoted by *B*, the fading factor of the transmission channel is denoted by *h*, *N* is the white Gaussian noise power, and *P* is the transmission power of the vehicular communication equipment. Therefore, according to the Shannon equation, the data transfer rate between any two communicating vehicles $v_{i}$ and $v_{j}$ at time *t* can be calculated as Equation (2):

(2) $$R_{V2V}=B_{V2V}\log_{2}\left(1+\frac{P_{V2V}10^{\frac{PL_{V2V}\left(t\right)}{10}}h^{2}}{N}\right).$$

In V2I communication mode, $PL_{V2I}$ denotes the path loss between the vehicle and the RSU, which is calculated in Equation (3):

(3) $$PL_{V2I}=d^{-\delta },$$

where *d* denotes the distance between the vehicle and the RSU, and δ denotes the path loss factor. The upstream and downstream links between vehicles and RSUs are Rayleigh flat fading channels. According to the Shannon equation, the data transmission rate between the vehicle and the RSU is

(4) $$R_{V2I}=B_{V2I}\log_{2}\left(1+\frac{P_{V2I}PL_{V2I}h^{2}}{N}\right).$$

### 3.2. Cost Model

Task data are denoted by a quad $<D,\alpha ,t^{max},\beta >$, where *D* denotes the task data size, $t^{max}$ denotes the latest completion time of the task, α denotes the number of cycles required by the CPU to complete the calculation of one bit of data, and β is the compression rate of the return value of the task.

The service vehicle *v* broadcasts its basic price $p_{v}^{base}$ and sets the differential price according to the priority of each task. The differential price is proportional to the priority of the task. To balance the load of all edge servers, we need to increase the computing power price of high-load servers and reduce the computing power price of low-load servers. The unit price of computing power charged by service vehicle *v* to task vehicle *u* is calculated using Equation (5):

(5) $$p_{v,u}=p_{v}^{base}+\omega_{p}\frac{D_{u}}{t_{u}^{max}}+\omega_{u}\left(\frac{f_{v}^{free}}{f_{v}^{max}}-1\right),$$

where $D_{u}$ denotes the data size of task vehicle *u*. $t_{u}^{max}$ denotes the latest completion time of task vehicle *u*. $\omega_{p}$ denotes the normalization factor of the differential price. $\omega_{u}$ is the normalization factor of server utilization, and $f_{v}^{free}/f_{v}^{max}-1$ is the server CPU utilization rate.

## 4. Computing Model and Utility Function

### 4.1. Computing Model

In this model, there are *M* service vehicles and *N* task vehicles. The task vehicle sends offloading requests to all service vehicles when task vehicle *u* decides its purchase strategy $f_{u}=\left(f_{u,1},f_{u,2},\ldots f_{u,m}\right)$. Task *T* is divided into M+1 subtasks $\left\{T_{0},T_{1},T_{2},\ldots ,T_{m}\right\}$. $T_{0}$ is computed locally, and $T_{1}∼T_{m}$ are offloaded to corresponding vehicles. After service vehicle *v* receives offloading requests of *N* task vehicles in a time slot, the service vehicle sends messages to all task vehicles if the computing resource value of total requests $f_{v}=\sum_{i=1}^{n}f_{i,v}$ is less than the maximum value $f_{v}^{free}$. If the computing resource value of total requests is greater than the maximum value and the excess part is $f_{v,cloud}=f_{v}-f_{v}^{free}=\sum_{i=1}^{n}f_{i,v,cloud}$, then the service vehicle purchases computing resources, and all the computing resources purchased from the cloud server are $f_{u,v,cloud}$. After the task vehicle receives the offloading message, it sends $\frac{f_{u,v}-f_{u,v,cloud}}{\alpha }$ size data to the service vehicle and $\frac{f_{u,v,cloud}}{\alpha }$ size data to the cloud server, where α denotes the number of cycles required by the CPU to complete the calculation of one bit of data. All task vehicles receive a message after the calculation is done, and the task is completed.

In a real environment, vehicles away from crossroads tend to offload entertainment tasks for comfort, and this type of task has a low priority. Vehicles near crossroads tend to offload tasks to ensure safe driving, and this type of task has a higher priority. $t^{max}$ denotes the latest completion time of the task:

(6) $$t^{max}=\gamma_{\mathrm{pr}}\left(x_{t}^{2}+y_{t}^{2}\right)D,$$

where $x_{t}$ and $y_{t}$ denote the abscissa and ordinate of the vehicle position at time *t*, respectively, the coordinate of intersection is (0,0), *D* denotes the task data size, $\gamma_{\mathrm{pr}}$ denotes the discount factor of task priority, and the task priority is proportional to the square of the distance from the task vehicle to intersection. We assume that task vehicle *u* needs to offload the subtask $T_{m}$ to service vehicle *v* and the computing resources that need to be purchased is $f_{u,v}$. The computing resources of a service vehicle is $f_{v}^{free}$. If $f_{u,v}<f_{v}^{free}$, then the service vehicle does not need to purchase additional computing resources from the cloud server, and it only uses the V2V communication mode. The completion time of the task is divided into three parts: upload time $t_{u,v}^{up}$, execution time $t_{u,v}^{exec}$ and feedback time $t_{u,v}^{down}$. Therefore, the completion time of the task can be calculated using Equations (7)–(10):

(7) $$t_{u,v}^{mec}=t_{u,v}^{up}+t_{u,v}^{exec}+t_{u,v}^{down},$$

(8) $$t_{u,v}^{up}=\frac{aD_{u}}{R_{V2V}},$$

(9) $$t_{u,v}^{exec}=\frac{a\alpha_{u}D_{u}}{f_{u,v}},$$

(10) $$t_{u,v}^{down}=\frac{a\beta_{u}D_{u}}{R_{V2V}},$$

where $a=f_{u,v}/f_{total}$ denotes offloading proportion, $f_{u}^{total}=f_{u}^{local}+\sum_{i=1}^{m}f_{u,i}$, $f_{u}^{local}$ denotes the computing power of the task vehicle, $R_{V2V}$ denotes the data transfer rate, $\alpha_{u}$ denotes the number of cycles required by the CPU to complete the calculation of one bit of data in service vehicle *v*, and $D_{u}$ is the task data size of task $T_{m}$. The completion time of the task can be expressed as Equation (11):

(11) $$t_{u}^{complete}=max\left\{t_{u}^{local},t_{u,1}^{mec},t_{u,2}^{mec},\ldots ,t_{u,m}^{mec}\right\},$$

where the local execution time $t_{u}^{local}=\left(\alpha D\right)/f_{u}^{local}$, according to Equation (9), Equation (12) can be derived as follows:

(12) $$t_{u,v}^{exec}=\frac{a\alpha_{u}D_{u}}{f_{u,v}}=\frac{\frac{f_{u,v}}{f_{u}^{total}}\alpha_{u}D_{u}}{f_{u,v}}=\frac{\alpha_{u}D_{u}}{f_{u}^{total}},$$

From Equation (12), we can conclude that all subtasks’ $t_{u,v}^{exec}$ have the same value. The completion time of task $t_{u}^{complete}$ is determined by the uploading time of each subtask and the computing resources purchased. Then, the task completion time $t_{u}^{complete}$ can be calculated as Equation (13):

(13) $$\begin{array}{c} t_{u}^{complete}=t_{u}^{local}+max\left\{t_{u,1}^{up}+t_{u,1}^{down},t_{u,2}^{up}+t_{u,2}^{down},\right.\\ \left.\ldots ,t_{u,m}^{up}+t_{u,m}^{down}\right\}, \end{array}$$

cloud server is introduced as backups. If all the computing resources purchased from the service vehicle are greater than free computing resources, subtasks $T_{1}∼T_{m}$ will be divided into $\left\{t_{1}^{mec},t_{1}^{cloud},t_{2}^{mec},t_{2}^{cloud},\ldots ,t_{m}^{mec},t_{m}^{cloud}\right\}$. The task time is divided into three parts if $f_{v}>f_{v}^{free}$. These parts are the local execution time, service vehicle execution time and cloud server execution time. The local execution time and service vehicle execution time have been expressed. The execution time of the cloud server using V2I communication can be calculated with Equation (14):

(14) $$t_{u,v}^{cloud}=t_{u,v}^{up}+t_{u,v}^{rsu,cloud}+t_{u,v}^{cloud\_exec}+t_{u,v}^{cloud,rsu}+t_{u,v}^{down},$$

upload time from task vehicle to the RSU is

(15) $$t_{u,v}^{up}=\frac{aD_{u}\left(f_{u,v}-f_{v}^{free}\right)}{R_{V2I}f_{u,v}},$$

upload time from the RSU to cloud server is

(16) $$t_{u,v}^{rsu,cloud}=\frac{aD_{u}\left(f_{u,v}-f_{v}^{free}\right)}{R_{LAN}f_{u,v}}+c\Delta t,c\in \left\{0,1,2\right\},$$

the value of $R_{LAN}$ is $10^{9}$, 10-GB Ethernet (10 GigE) was used between RSUs in proposed model. Due to the distance between two RSUs is too close, the transfer time is denoted as a constant Δt. c∈{0,1,2} denotes the hop counts between two RSUs. The execution time on cloud servers is calculated as Equation (17):

(17) $$t_{u,v}^{cloud\_exec}=\frac{a\alpha_{u}D_{u}}{f_{u,v}-f_{v}^{free}},$$

transfer time from cloud server to RSUs is

(18) $$t_{u,v}^{cloud,rsu}=\frac{a\beta_{u}D_{u}\left(f_{u,v}-f_{v}^{free}\right)}{R_{LAN}f_{u,v}}+c\Delta t,$$

feedback time of the task result is

(19) $$t_{u,v}^{down}=\frac{a\beta_{u}D_{u}\left(f_{u,v}-f_{v}^{free}\right)}{R_{V2I}f_{u,v}},$$

In summary, the task completion time of the task vehicle can be calculated as Equation (20):

(20) $$t_{u,v}^{complete}=\max\left\{t_{u}^{local},t_{u,v}^{mec},t_{u,v}^{cloud}\right\}.$$

### 4.2. Utility Function

We hope that the offloading task be completed within reasonable costs. Therefore, the optimization goals of the task vehicle are the task time, task costs, and task rewards. The utility function of the task vehicle can be calculated as Equation (21):

(21) $$U_{task}=aU_{time}-bU_{pay}+cU_{success},$$

where a+b+c=1, *a* denotes price sensitive factor, *b* denotes delay sensitive factor, and *c* denotes task success sensitive factor. $U_{time}$ denotes the satisfaction caused by time savings, and the more computing power the task vehicle purchases, the larger the value of $U_{time}$. $U_{pay}$ denotes the satisfaction produced by task costs. The value of the task costs is larger, and the value of $U_{pay}$ is smaller. $U_{time}$ can be calculated as Equation (22):

(22) $$U_{time}=\gamma_{1}\mathrm{Pr}\ln\left(1+t^{save}\right),$$

where $t^{save}$ denotes the value of time saved by an offloading task. $\gamma_{1}$ denotes the normalization coefficient. As the value of $t^{save}$ increases, the utility of vehicles with higher offloading priority become higher. $t^{save}$ can be calculated as Equation (22):

(23) $$t^{save}=t^{all\_local}-\max\left\{t^{local},t^{mec},t^{cloud}\right\},$$

where, $t^{all\_local}$ denotes the completion time that all tasks compute locally. $U_{success}$ denotes the task reward, and it is a constant greater than 0. *c* is a positive number. If the task is completed within the latest completion time, then the value of *c* is *c*. Otherwise, the value of *c* is −c. Last, the utility function of the task vehicle can be derived as follow equation:

(24) $$\begin{array}{cc} U_{task} & =a\gamma_{1}\mathrm{Pr}\ln\left(1+t^{all\_local}-t^{complete}\right)-b\left(\gamma_{2}{\left(\sum_{i=1}^{m}f_{t,i}p_{i,t}+Eb_{local}\right)}^{y}\right)+cU_{success}\\ \; & =a\gamma_{1}\frac{D}{t^{\max}}\ln\left(1+\frac{\alpha D}{f_{local}}-\max\left\{\frac{\alpha D}{f_{total}},t_{1}^{mec},\ldots ,t_{m}^{cloud}\right\}\right)\\ \; & -b\left(\gamma_{2}{\left(\sum_{i=1}^{m}f_{t,i}p_{i,t}+Eb_{local}\right)}^{y}\right)+cU_{success}\\ \; & s.t.:0\leq a\leq 1,0\leq b\leq 1,0\leq c\leq 1,a+b+c=1\\ \; & \gamma_{1}>0,\gamma_{2}>0,D>0,\alpha >0,\beta >0,y>0,P^{\min}\leq p_{i,L},R_{success}>0\leq P^{max},i\in V_{s}\\ \; & c=\left\{\begin{array}{c} c,if\max\left\{\frac{\alpha D}{f_{total}},t_{1}^{mec},\ldots ,t_{m}^{cloud}\right\}\leq t^{max}\\ -c,if\max\left\{\frac{\alpha D}{f_{total}},t_{1}^{mec},\ldots ,t_{m}^{cloud}\right\}>t^{max} \end{array}\right., \end{array}$$

where, $U_{time}$ is an increasing function of $t^{save}$. However, considering user’s economic factor, the marginal utility of $U_{time}$ decreases when the task vehicle continuously purchases computing resources to increase $t^{save}$. Meanwhile, the satisfaction of task vehicles is a subtraction function of $f_{t}=\sum_{i=1}^{m}f_{t,i}$, $f_{t}$ is the total computing resources a task vehicle buys from all service vehicles. $U_{pay}=\gamma_{2}{\left(p_{\mathrm{pay}}+Eb_{local}\right)}^{y}$. $Eb_{local}$ is the electricity consumption for local computing. $p_{i,t}$ denotes the unit price of computing resources sold by service vehicles. $\gamma_{1}$ is the time discount factor of utility function. $\gamma_{2}$ is expenditure discount factor.

The revenue of the service vehicle is the task vehicle cost minus the electricity cost for computing and the cost for purchasing resources from the cloud server. To ensure that $U_{service}$ is always greater than 0, we assume that $p_{s,t}\geq P_{cloud}$ and cloud server resources are unlimited. The utility function of service vehicle *v* can be calculated as Equation (25):

(25) $$U_{service}=\sum_{i=1}^{n}f_{i,v}p_{v,i}-ek\sum_{i=1}^{n}f_{i,v}^{2}\frac{\alpha_{i}D_{i}}{f_{i}^{total}}-P_{cloud}\sum_{i=1}^{n}f_{i}^{cloud},$$

$P_{u,v}$ denotes the unit price of computing resources sold by the service vehicle. $P_{cloud}$ denotes the unit price of computing resources sold by the cloud server. *e* is unit price of electricity. We used $P_{cpu}$ to denote the power of equipment, according to [32], $P_{cpu}=kf^{2}$, where *k* is CPU energy coefficient.

## 5. Stackelberg–MADDPG Task Offloading Algorithm

We propose an offloading strategy based on multi-agent reinforcement learning. In the traditional multi-agent deep deterministic policy gradient(MADDPG) algorithm, agents lack a hierarchical relationship and make decisions at the same time. To solve this problem, we propose a Stackelberg–MADDPG algorithm with a master–slave relationship. The state space *S*, action space *A* and reward function *r* of the agent are defined as follows:

The state space of the leader agent at time *t* is defined as Equation (26):

(26) $$s_{t}^{leader}=\left\{i\left(t\right),P_{cloud}\left(t\right),f_{free}\left(t\right),u\left(t\right)\right\},$$

where $P_{cloud}\left(t\right)$ denotes the unit price of the cloud server at time *t*. $f_{free}\left(t\right)$ denotes the computing resources available to the service vehicle at time *t*, and u(t) denotes the resource utilization of each service vehicle at time *t*. i(t) denotes the vehicle information set at time *t* including the position, speed, acceleration, etc.

The action space of the leader agent at time *t* is defined as Equation (27):

(27) $$a_{t}^{leader}=\left\{p\left(t\right)\right\},$$

where p(t) denotes the unit price of computing power at time *t*. The state space of each follower agent is defined as Equation (28):

(28) $$s_{t}^{follower}=\left\{i\left(t\right),P_{service}\left(t\right),T\left(t\right),R\left(t\right)\right\},$$

where $P_{service}\left(t\right)$ denotes the decision set of the leader agent at time *t*. T(t) denotes the parallel task set at time *t*, including the task size, latest completion time, time-sensitive factor, price-sensitive factor, etc. R(t) denotes the data transmission rate set of each V2V and V2I link at time *t*. The action space of each follower agent is defined as Equation (29):

(29) $$a_{t}^{follower}=\left\{f\left(t\right)\right\},$$

where f(t) denotes the set of computing resources purchased by the task vehicle from service vehicles. The reward function for the follower is calculated as Equation (30):

(30) $$r\left(s_{t}^{follower},a_{t}^{follower}\right)=U_{task}.$$

The reward function for the leader is calculated as Equation (31):

(31) $$r\left(s_{t}^{leader},a_{t}^{leader}\right)=U_{service}.$$

The total reward of each agent, which is the objective function of MADDPG, can be calculated as Equation (32):

(32) $$J\left(\theta \right)=\max E\left[\sum_{t=0}^{T}\gamma^{t}r\left(s_{t},a_{t}\right)\right].$$

Algorithm 1 shows the pseudo-code of the Stackelberg–MADDPG.
**Algorithm 1:** Stackelberg-MADDPG algorithm in Internet of Vehicles
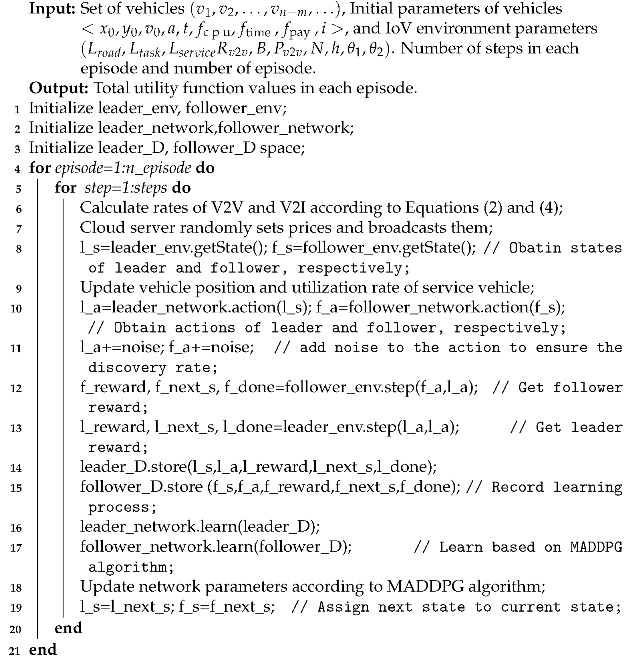


**Figure 3:**
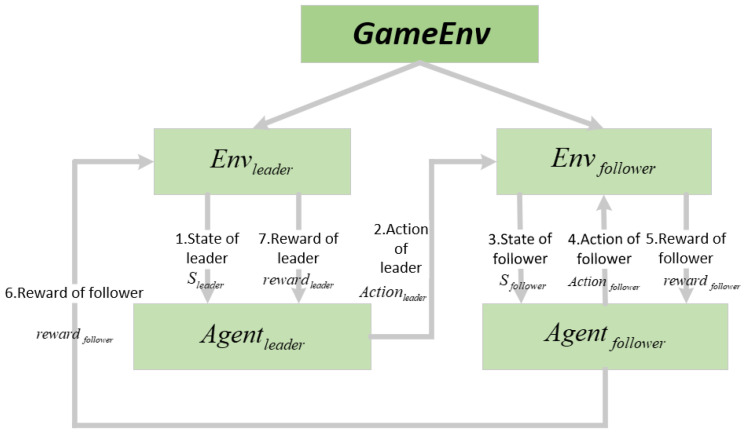
Stackelberg–MADDPG algorithm model.

The Stackelberg–MADDPG algorithm model is shown in Figure 3.

## 6. Simulation and Analysis

### 6.1. Experimental Settings

Our experimental environment is Python 3.7, PyTorch 1.7.1, CUDA 11.0, Intel (R) Core (TM) I7-9750H 2.60-GHZ CPU, NVIDIA GTX1650 GPU, 8GB RAM and Windows 10. In our experiments, there are five service vehicles and 20 task vehicles. Each service vehicle can provide 5 Ghz of computing resources. Task vehicles compete for limited computing resources. The price sensitivity coefficient *a*, delay sensitivity coefficient *b*, task success sensitivity coefficient *c* and initial coordinate $\left(x_{t},y_{t}\right)$ of each vehicle are randomly generated. In a real scenario, communication parameters are determined by the vehicle and the edge equipment; Task parameters are determined by users’ computing requirements and external environment; The parameters in reinforcement learning algorithm can be determined by continuous debugging.

There are 25 agents in our experiment. Each agent has two networks and two target networks: Actor network, Critic network, Actor target network, and Critic target network. Actor network and Critic network adopt four-layer fully connected network. The first layer of Actor network and Critic network is the input layer. The number of neurons for Actor network is equal to the dimension of the state vector. The number of neurons for Critic network is equal to the dimension of the state vector plus the dimension of action vector. The number of neurons in the second layer of Actor network and Critic network is 256, and the number of neurons in the third layer is 128. The fourth layer is output layer. Since the Actor network outputs the actions of agents, the number of neurons in the output layer is the dimension of the agent’s action vector. The output layer of Critic network has one neuron. We adopt the Adam optimizer as the training optimizer. When updating the Actor network and Critic network by the stochastic gradient method, the learning rate of the Actor network is 0.0001, that of the critic network is 0.001, the reward discount is 0.9, the batch size is set to 64, the size of the experience playback pool is 106 and the number of training rounds is 32. The number of training rounds for the agent is determined according to the complexity of the environment and is generally 2000–4000 rounds. The parameters and values in the Internet-of-Vehicles environment are listed in Table 1.

### 6.2. Results and Analysis

**Figure 4:**
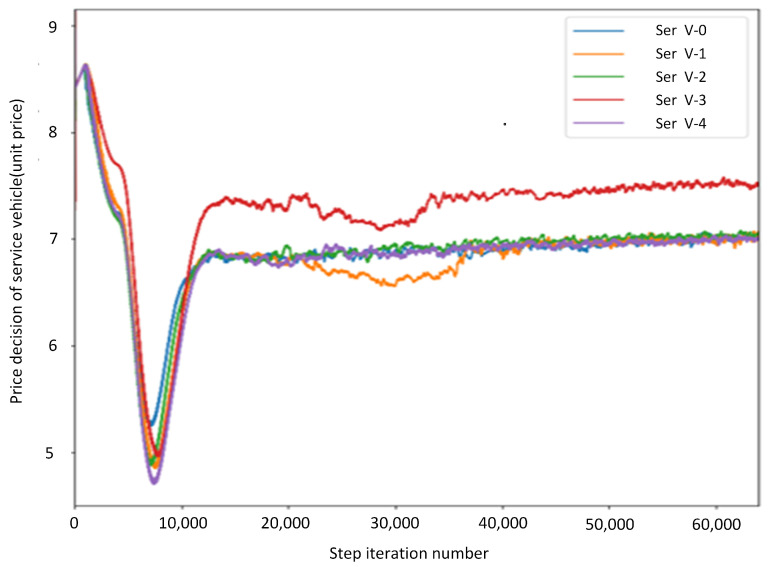
Price decision for service vehicles.

Figure 4 shows the iterative convergence of the service vehicle price decision. It fluctuates greatly before the service vehicle price decision converges, mainly because the algorithm needs all the information of the leader during training. In other words, the proposed algorithm relies on full cooperation among all leaders to achieve the goal of global optimization. Service vehicle 3 is closer to other task vehicles and has more computing resources. Offloading to service vehicle 3 can save more time and improve users’ satisfaction with time delay. Therefore, setting a higher price can get more revenue for service vehicle 3. After convergence, except for service vehicle 3, the decision curves of all service vehicles intersect near the price of 6.8 and fluctuate slightly with changes in the environment. It can be concluded that when the change in the environment is small, the price of 6.8 is the equilibrium point of all service vehicles. At this time, a change in strategy does not increase the utility value.

**Figure 5:**
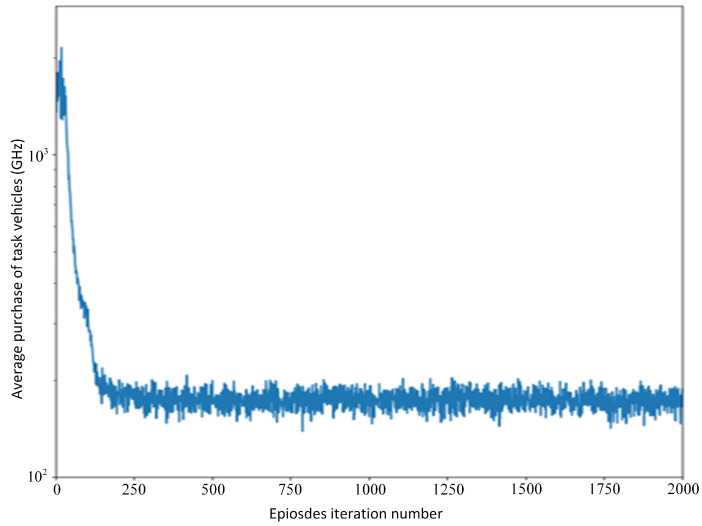
Average purchase for task vehicles.

Figure 5 shows the average purchase decision of task vehicles. The purchase of the task vehicles drops sharply before 250 episodes and then converges gradually. At this time, the decision of each task car reaches the optimal level.

**Figure 6:**
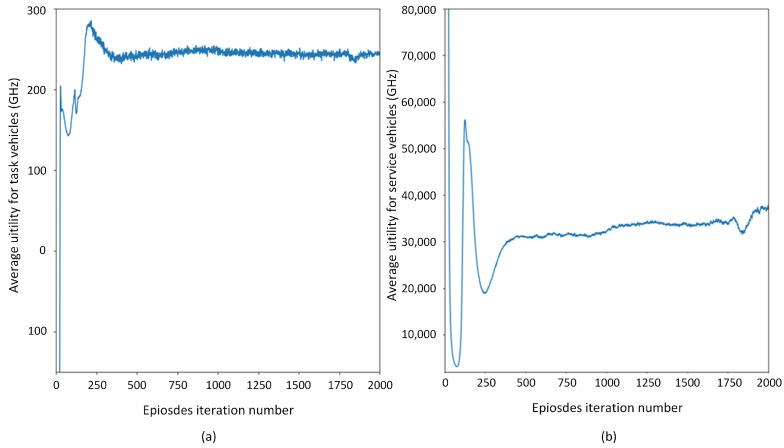
Changes in average utility.(**a**) Task vehicles, (**b**) service vehicles.

Figure 6 shows the changes of leader’s price strategy and follower’s purchasing strategy when the unit price is $4\times 10^{-9}$. The leader’s strategy and follower’s strategy converge after 1200 episodes and 250 episodes, respectively. In 0∼280 episodes, the leader’s price strategy and the follower’s purchasing strategy continues to decrease. Reduced purchasing strategies and lower prices lead to lower time delay and lower expenses; then, $U_{time}$ increases and $u_{pay}$ decreases. As a result, the average reward for followers $U_{task}$ increases. Leaders’ rewards decrease with lower prices and fewer purchases. In Figure 4, we can see that the price touches the bottom at 9000th steps (281th episodes). Meanwhile, the leader’s average utility reaches local minimum value and the follower’s average utility reaches the maximum value. Followers’ purchasing strategies tend to stabilize. Leaders learn that utility continues to go down and starts to increase prices. Then, $u_{pay}$ increases, which leads to a sharp drop in followers’ average rewards and an increase in leaders’ rewards. The growth rate of the leader’s utility tends to 0 when the leader’s price strategy increases from 4.6 to 6.7. In 14,000∼60,000 steps (420∼2000 episodes), leaders’ prices increase slowly, and followers adjust their strategies to keep rewards steady. The service vehicle price decision, task vehicle purchase decision, service vehicle reward and task vehicle reward converge after roughly 1200 iterations. Therefore, the Nash equilibrium solution in the current Internet-of-Vehicles environment is as follows: the price is approximately 6.8 units per 1 Hz, and the task vehicles purchase approximately 110 GHz each time.

**Figure 7:**
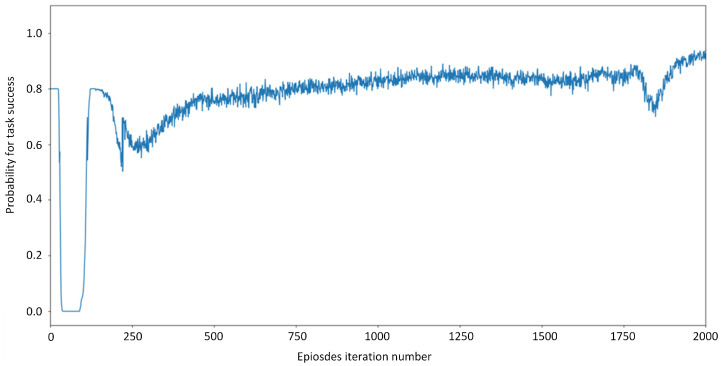
Curve for task success rate.

Figure 7 shows the curve for task success rate. During 0∼300 iterations, the task success rate fluctuates in 0∼80%. After 500 iterations, the task success rate is stable at 80%. During 500∼1250 iterations, the task success rate increased slowly from 80% to 85%. After that, the task success rate fluctuates 70∼85% up to 1800 iterations. After 1800 iterations, the task success rate rocketed to 90% and then stabilized. Why can the task success rate not rise above 95%? First, as a,b,c are generated randomly, the value of task success sensitive factor *c* could be small, and the task cannot be completed within the specified time. Second, the task data size is randomly generated from 800 MB to 1300 MB, and the task completion delay within 1∼5 s. Assume that the data size of a task is 1300 MB and the task completion delay is 1 s. If enough computing resources are purchased, the task vehicle’s utility will reach −20, where the gains do not make up for the losses. Therefore, the proposed offloading algorithm can maintain a high task success rate and make a trade-off between utility and success rate.

**Figure 8:**
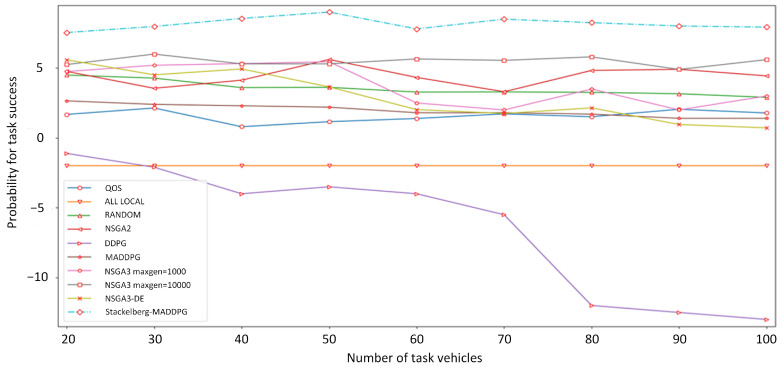
Average utility curve of task vehicles with different algorithms.

To further verify the performance of this algorithm, several typical algorithms are compared in Figure 8:

1. Non-dominated Sorting Genetic Algorithms (NSGA): To obtain a Pareto optimal solution of purchase strategy and price strategy, both purchase strategy and price strategy are determined by the algorithm simultaneously. We select the results and running time of NSGA-II, NSGA-III and NSGA-III-DE algorithms. For NSGA-IIIS, to compare the influence of genetic algebra on the results, we set the maximum genetic algebras (maxgen) to 1000 and 10,000, respectively. NSGA-III-DE combines the advantages of NSGA-III and differential evolution (DE) algorithm, which optimizes the generation of offspring.
2. Deep deterministic policy gradient (DDPG) algorithm: DDPG is a single agent reinforcement learning algorithm. The service vehicles and task vehicles are abstracted as an agent, and they make decisions simultaneously. The optimization objective is the weighted sum of service vehicle and task vehicle rewards.
3. MADDPG algorithm: The service vehicles and task vehicles are abstracted as multiple agents, and each agent makes strategies at the same time. The optimization objective is to maximize the cumulative rewards of each agent.
4. Random algorithm: Purchasing decision, local offloading ratio and edge server offloading ratio are randomly generated.
5. Quality of service (QoS) algorithm: All the tasks are equally allocated to service vehicles. Task vehicles purchase the maximum computing resources to save time delay.
6. All-Local algorithm: All tasks are executed locally without offloading.

In the change curve of the average utility of task vehicles, the utility of the DDPG algorithm decreases rapidly with an increase in the number of task vehicles. The QoS algorithm [22], all-local algorithm and random algorithm are mediocre. Both the QoS algorithm and all-local algorithm are extreme and cannot achieve a compromise between delay, payment and task success or failure. The Stackelberg–MaddPG algorithm, NSGA-III algorithm [33] and NSGA-III-DE algorithm exhibit excellent performance. The utility value of the task vehicle does not decrease with the increase in the number of task vehicles, and the utility value is higher than that of other algorithms. The Stackelberg–MaddPG algorithm has a 25% higher average utility than the NSGA-III algorithm and 1.5 times the average utility value of the NSGA-III-DE algorithm.

As the Internet-of-Vehicles environment is time sensitive, the offloading system needs to make offloading decisions in a very short time. Table 2 shows the execution and training time of this algorithm and other algorithms. In Table 2, the QoS algorithm, all-local algorithm and random algorithm do not need to be trained, and the algorithm execution time is negligible. Several NSGA algorithms also do not require training, but the execution time of the algorithm is too long, even up to hundreds of seconds, which is certainly not suitable for the time-sensitive networking environment. Although the DDPG algorithm, MADDPG algorithm and Stackelberg–MADDPG algorithm take a long time to train, after training, they take only 50 milliseconds to calculate an offloading decision.

## 7. Conclusions

In view of the insufficient computing power of service vehicles in the Internet of Vehicles, we use the computing power of a cloud server as a supplement. Based on the idea of mobile edge computing, we propose a task offloading scheme of local-edge-cloud collaborative computing in the IoV environment. The simulation results show that our Stackelberg–MADDPG algorithm performs faster than other algorithms. It improves the success rate of task execution while effectively achieving a balance between task vehicle delay and cost. However, the proposed system could be further improved. First of all, there is only one or two roads in this model, which is too simple and far from real roads. Real roads simulation will be used for modeling in the future. In addition, vehicles are selected as edge servers in this paper. In fact, tasks could be offloaded to other devices. Then, when modeling in real scenarios, the price model needs to be taken more factors into account. Many parameters are only experimentally desirable. We can also set a smaller price sensitive factor to reduce the effect of price on utility. In the real world, parameters will be changed according to the environment. However, the main purpose of this paper is to study the offloading method in IoV. We will further improve the price model in the future work, such as introducing a business model to make our experiment more realistic. Finally, we will study how task vehicles reserve computing resources of service nodes in advance and plan offloading paths according to pre-set destinations.

## Figures and Tables

**Table 1:** Experimental parameters of Stackelberg–MADDPG offloading algorithm.

Parameter	Definition	Value
$L_{width}$	width of road	20 m
$L_{road\_h}$	transverse length of road	200 m
$L_{road\_v}$	longitudinal length of road	200 m
$R_{V2V}$	V2V communication distance	50 m
$P_{V2V}$	V2V communication power	20 W
$P_{V2I}$	V2I communication power	30 W
*B*	communication bandwidth	3.5 GHz
$f_{\mathrm{local}}$	computing power of task vehicle	2.2 GHz
$f_{s}$	computing power of service vehicle	2∼5 GHz
*N*	white Gaussian noise power	$2.5*10^{-13}$ W
*h*	fading factor	4
$\omega_{p}$	differential price factor	$7*10^{-11}$
$\omega_{u}$	server utilization normalization factor	$2*10^{-9}$
*a*	price sensitive factor	0∼1
*b*	delay sensitive factor	0∼1
*c*	task success sensitive factor	0∼1
*D*	task data size	1000∼1700 MB
$U_{success}$	reward for mission success	20
$\gamma_{1}$	time discount factor of utility function	10
$\gamma_{2}$	expenditure discount factor	0.15
$\gamma_{pr}$	task priority discount factor	0.16667
$\gamma_{t}$	discount factor of task vehicle	0.26
$\gamma_{s}$	discount factor of service vehicle	0.0001
$P_{cloud}$	computing power price of cloud sever	$1.5*10^{-9}$
u_p	unit price of computer power	$4*10^{-9}$
*e*	unit price of electricity	$2.78*10^{-6}$
a	acceleration	0∼1
$v_{0}$	initial velocity	30∼50 km/h

**Table 2:** Execution time and training time of different task offloading strategy algorithms.

Task Offloading	Algorithm Execution	Algorithm Training
Decision Algorithm	Time (s)	Time (h)
QoS	0.0056	0
All-Local	0.0032	0
Random	0.0068	0
NSGA-III-DE	973	0
NSGA-III (maxgen = 1000)	62	0
NSGA-III (maxgen = 10,000)	1061	0
NSGA-III	467	0
DDPG	0.040	3
MADDPG	0.043	9
Stackelberg-MADDPG	0.052	20
